# Effectiveness of dentin pre-treatment on bond strength of two self-adhesive resin cements compared to an etch-and-rinse system: an *in vitro* study

**DOI:** 10.7717/peerj.11736

**Published:** 2021-10-26

**Authors:** Milad Hammal, Zdeněk Chlup, Tomáš Ingr, Ján Staněk, Radek Mounajjed

**Affiliations:** 1 Institute of Dentistry and Oral Sciences, Palacký University, Olomouc, Czech Republic; 2 Academy of Science of the Czech Republic, Institute of Physics of Materials, Brno, Czech Republic; 3 Faculty of Science, Department of Experimental Physics, Palacký University, Olomouc, Czech Republic; 4 DCM Clinic, Hradec Kralove, Czech Republic

**Keywords:** Adhesion, Bond strength, Dentin surface pre-treatment, Lithium disilicate, Self-adhesive resin cements

## Abstract

**Statement of problem:**

It has been shown that selective etching improves the bond strength of some self-adhesive resin cements to enamel. The same has yet to be determined with dentin pre-treatment.

**Purpose:**

To evaluate the tensile bond strength of two self-adhesive resin cements after two dentin surface pre-treatments, and also to analyze the cement/dentin interface.

**Material and Methods:**

One hundred and twelve human third molars were extracted. The teeth were distributed into seven groups (*n* = 16). Maxcem Elite Chroma (MAX) (Kerr, Scafati, Italy) and Relyx U200 (RLX) (3M ESPE, Neuss, Germany) were used without pre-treatment or with two dentin pre-treatments (polyacrylic acid or phosphoric acid). A conventional etch-and-rinse (EAR) luting cement, NX3 Nexus (NX3) (Kerr, Scafati, Italy), was used as an external control group. Before testing, all specimens were stored in distilled water for 24 hours. Three specimens from each group were prepared for scanning electron microscopy observation (SEM). A tensile bond strength test (TBS) was performed for the remaining samples. The data were statistically analyzed using the Kruskal–Wallis test and Pairwise comparisons using the Wilcoxon rank sum test.

**Results:**

MAX without pre-treatment and with phosphoric acid etching attained statistically similar bond strengths to NX3 (*P* > 0.05). There was a statistical difference (*P* = 0.00488) between RLX without pre-treatment (5.62 MPa) and NX3 (10.88 MPa). Phosphoric acid pre-treatment increases the bond strength values of RLX to a strength that is comparable to NX3 (*P* > 0.05). The lowest tensile bond strength (TBS) was attained after the application of polyacrylic acid with MAX (1.98 MPa). No statistical differences were found between the RLX bond strength values after polyacrylic acid treatment and RLX without pre-treatment or NX3 (*P* > 0.05). SEM observations disclosed an enhanced potential of the self-adhesive cements to infiltrate into dentin tubules and form resin tags when applied after phosphoric acid pre-treatment. The failure mode was dominantly adhesive.

**Conclusions:**

On dentin, the self-adhesive resin cement MAX might be an effective alternative to conventional resin cement. Etching the dentin with phosphoric acid does not have a negative effect on the bond strength of MAX to dentin. On the other hand, phosphoric acid improved the bond strength of RLX when compared to EAR cement.

## Introduction

With the improvements in adhesive dentistry, resin cements have played an important role in prosthodontic dentistry (Pavan et al., 2010). Resin cements provide several advantages when compared to conventional luting systems (such as zinc phosphate and glass ionomer), which include: high bond strength, minimal solubility in the oral environment, stability, a durable tooth-restoration interface, and a minimum need for the reduction of dental tissues (Han et al., 2007). The conventional resin cements are based on the etch-and-rinse (EAR) technique, which is a multi-step system that is considered complex and technique-sensitive. The need to rinse the acidic gel and the risk of over-etching or over-drying may impair the bonding efficacy and compromise the survival rates of the bonded restoration (Pashley et al., 2011). For these reasons, many self-adhesive resin cements have been introduced onto the dental market. These cements include the application of the adhesive and cement at the same time, in addition to eliminat the need for tooth structure pre-treatment (De Munck et al., 2004). The self-adhesive cements have been indicated to lute all metallic-base materials and ceramic and indirect composite restorations (Jaberi Ansari & Kalantar Motamedi, 2014). However, the cementation of such restorations using these simplified cements on smear layer-covered dentin still remains a concern (Mazzitelli et al., 2010). These self-adhesive luting agents are composed of aqueous mixtures of acidic functional monomers such as 2-hydroxyethyl methacrylate (HEMA), urethane dimethacrylate (UDMA), and 10-methacryloxydecyl dihydrogen phosphate (MDP), with a pH higher than that of phosphoric acid gel (Parra Lozada & Garzón Rayo, 2012; Peçanha et al., 2021). In spite of the manufacturer’s claim that no pre-treatment for dental tissues is necessary, nevertheless, the bond strength on enamel, for example, has been considered inadequate in comparison to that achieved with the EAR technique, which requires an adhesive system (Sekhri, Mittal & Garg, 2016). In relation to dentin, some studies showed that the performance of self-adhesive resin cements is comparable to that of etch-and-rinse systems on coronal dentin. Comparison of the shear bond strength of self-adhesive resin cements to enamel and dentin with different protocol of application (Moghaddas et al. 2017). Rodrigues et al. (2015), while other studies showed opposite results, the bond strength to dentin with self-adhesive cements being significantly lower (De Angelis et al., 2011; Lührs et al., 2010; Viotti et al., 2009). For improved bond strength to enamel, selective etching with phosphoric acid (H3PO4) has been proposed. However, this carries the risk of contaminating neighboring dentin surfaces with the phosphoric acid (Burrer et al., 2020). Relating to this issue, some studies showed that etching the dentin with phosphoric acid reduces the bond strength (De Munck et al., 2004; Hikita et al., 2007). In contrast, another study showed higher bond strength (Pisani-Proença et al., 2011). This makes the phosphoric acid placed on dentin still a controversial issue.

It has also been suggested to apply a weak acid such as polyacrylic acid (PAA), to improve the bond strength of self-adhesive cements to dentin (Pavan et al., 2010). Polyacrylic acid is a mild conditioning agent employed for cavity cleansing and surface conditioning in glass ionomer restorations. However, some worries exist regarding its application times and concentrations. Such factors might interfere with the bonding performance. Dominant adhesive failures between a resin-modified glass ionomer cement (RMGIC) and resin composite have been reported when a PAA was used on dentin covered by a smear layer (Sauro et al., 2018).

Therefore, the purpose of this study was to evaluate the effectiveness of dentin pre-treatment with both PAA and/or H3PO4 using two self-adhesive resin cements compared to an etch-and-rinse system on the tensile bond strength between lithium disilicate restorations and dentin, and to evaluate the cement/dentin interfaces using scanning electron microscopy analysis. The null hypothesis is that the dentin pre-treatment with both acids (H3PO4 and/or PAA) does not affect the bond strength of self-adhesive resin cement.

## Materials and Methods

One hundred and twelve caries-free third molars recently extracted from patients aged 20–30 years because of pericoronitis were collected. Ethics approval number 80/21 from the Ethics Committee at Palacky University and verbal consent of the donors were obtained. The teeth were then cleaned with an ultrasonic scaler and stored in a 10% formalin solution (HistoFOR BFS-L1; Pro-charitus.r.o, CZ), for one week after extraction (Mounajjed et al., 2018), and then the teeth were kept in distilled water until use. The teeth were tested within a maximum of one month after extraction.

The roots of the teeth were embedded in auto-polymerized acrylic resin (Spofacryl™; SpofaDentala.s, Jičín, CZ) to facilitate handling during the cutting and testing procedures.

Two self-adhesive dual-cure cements, Maxcem Elite Chroma (Kerr, Scafati, Italy) and Relyx U200 (3M ESPE, Neuss, Germany), were used. A conventional resin dual-cure cement, NX3 Nexus (Kerr, Scafati, Italy) (Table 1), which requires the application of an adhesive, was also used as an external control group, since the EAR system is still considered as the gold standard for dental adhesion (Parra Lozada & Garzón Rayo, 2012).

**Table 1:** Shows the composition, batch number, and manufacturer of materials used in this study.

Material	Manufacturer Lot number	Composition	Application
Relyx U200	(3M ESPE, Neuss, Germany) 4957491	Base paste: glass powder treated with silane, 2- propenoic acid, 2-methyl 1,10- (1-[hydroxymetil]-1,2- ethanodlyl) ester dimethacrylate, triethylene glycol dimethacrylate (TEGDMA), silica treated silane, glass fiber, sodium persulfate and per-3,5,5-trimethyl hexanoate t-butyl; catalyst paste: glass powder treated with silane, substitute dimethacrylate, silica-treated silane, sodium ptoluenesulfonate, 1-benzyl-5- phenyl-acid barium, calcium, 1,12-dodecane dimethacrylate, calcium hydroxide, and titanium dioxide	Apply the cement after mixing on the ceramic surface; seat the restoration gently onto the preparation allowing the cement to flow from all sides, then press. All samples were put under a static load, waiting for 60 s; during this time we clean the excess cement, then light cure for 20 s from each side (60 s in total)
Maxcem Elite Chroma	(Kerr, Scafati, Italy) 7205841	HEMA, GDM, UDMA, 1,1,3,3- tetramethylbutyl hydroperoxide TEGDMA, fluoroaluminosilicate glass, GPDM, barium glass filler, fumed silica (69 wt %))	Apply the cement after mixing on the ceramic surface; seat the restoration gently onto the preparation allowing the cement to flow from all sides, then press. All samples were put under a static load, waiting for 60 s; during this time we clean the excess cement, then light cure for 20 s from each side (60 s in total)
Nexus NX3 dual-cure	(Kerr, Scafati, Italy) 7233567	Uncured methacrylate ester monomers, HEMA, PTU, CHPO, free tertiary amines and benzoyl peroxide, inert mineral fillers, titanium dioxide, radiopaque agent, and pigments	Apply the cement after mixing on the ceramic surface; seat the restoration gently onto the preparation allowing the cement to flow from all sides, then press. All samples were put under a static load, waiting for 60 s; during this time we clean the excess cement, then light cure for 20 s from each side (60 s in total)

### Dentin specimen preparation

The crowns of the teeth were cut perpendicularly to the long axis of the tooth with a low-speed diamond saw (IsoMet; Buehler, Lake Bluff, IL, USA) under copious water to expose a flat, middle third dentin surface. The ground dentin surfaces were observed under an optic microscope to verify complete enamel removal. A standardized smear layer was achieved by grinding the flat dentin surfaces using 320-grit silicon carbide paper with a single-wheel grinder and polisher (Saphir 550, Metalco Testing s.r.o, Roztoky u Prahy, CZ) for one minute under continuous water irrigation to simulate the creation of a smear layer that would be created clinically by a red diamond bur (Kharouf et al., 2019). The teeth were kept in distilled water during and between all the experimental procedures. The teeth were randomly divided into seven groups consisting of 16 teeth each.

Group 1 (NX3) (the external control group), the dentin etched for 10 s with Kerr Gel etchant 37.5% phosphoric acid (Kerr, Scafati, Italy), then was thoroughly washed using a water spray for at least 30 s, then gently air-dried for 5 s. Primer (OptiBond FL, Kerr, Scafati, Italy) was applied twice, followed by air-drying for 15 s, then adhesive (OptiBond FL, Kerr, Scafati, Italy), then air-drying for 15 s, and after that NX3 Nexus dual-cure resin cement was applied.

Group 2 (MAX-no): no dentin pre-treatment, Maxcem was applied according to the manufacturer’s instructions.

Group 3 (MAX-PAA): after the application of 25% polyacrylic acid (Ketac Conditioner; 3M ESPE, Seefeld, Germany) for 15 s, the acid was thoroughly washed using a water spray for at least 30 s, then Maxcem was applied.

Group 4 (MAX-HPO): after the application of 37.5% phosphoric acid for 10 s, the acid was thoroughly washed using a water spray for at least 30 s, then Maxcem was applied.

Group 5 (RLX-no): no dentin pre-treatment, Relyx was applied according to the manufacturer’s instructions.

Group 6 (RLX-PAA): after the application of 25% polyacrylic acid (Ketac Conditioner; 3M ESPE, Seefeld, Germany) for 15 s, the acid was thoroughly washed using a water spray for at least 30 s, then Relyx was applied.

Group 7 (RLX-HPO): after the application of 37.5% phosphoric acid for 10 s, the acid was thoroughly washed using a water spray for at least 30 s, then Relyx was applied.

Before the bonding procedure in all groups, the moisture in the dentin was moderately removed with short, moderate blasts of air, leaving a bright surface without any fluid movement.

### Preparation of ceramic blocks

The sample design (5 mm in diameter and 10 mm in height) was prepared to be printed out by a Straumann P series Rapidshape 3D printer using Detax Freeprint resin for the digital production of the cast pattern (100% residue-free burning), then lithium disilicate-based ceramic cylinders (IPS e.max Press; IvoclarVivadent, Schaan, Liechtenstein) were made with the hot-pressing technique. The resin samples were attached to wax sprues and invested in flasks using an investment material (Pressvest speed, Ivoclar vivadent, Liechtenstein), before burning the resin out in a furnace (EP 600; IvoclarVivadent, Schaan, Liechtenstein). Afterwards, e.max press ingots were heat-pressed into the space created by the burned resin. The ceramic samples, when cooled, were removed from the flasks, sprues were cut. Then the samples were removed also from the investment material (IPS^®^ Press Vest InvestmentMaterial; IvoclarVivadent, Schaan, Liechtenstein), smoothed and polished according to the manufacturer’s instructions. Before the cementation process, the ceramic samples were etched with 9% hydrofluoric acid (Porcelain etch; Ultradent Products, Inc, Cologne, Germany) for 20 s, then rinsed with air/water for 30 s. The samples were then immersed in 96% alcohol (Ethanolum 96%; Fagron. CZ) and then subsequently in an ultrasonic bath (ZZlinker,LK-D32, China) for 5 min (Mounajjed et al., 2018), for better removal of the product residues after acidic conditioning.

After that, a silane (Ceramic silane; Ultradent Products, Inc, Cologne, Germany) was applied to the etched ceramic surfaces two times with a microbrush.

### Cementation procedures

The ceramic samples were cemented to the dentin surfaces using the different cements, according to the mentioned application in the Table 1. The bonding of the ceramic samples using the resin cements was achieved under a static load (250 g) until complete setting to simulate and standardize finger pressure (Ferro et al., 2016).

The excess cement was removed with a microbrush and scalpel blade. The samples were light-cured with a polymerization light unit (VALO; Ultradent Products, Inc, Cologne, Germany) for 60 s from all sides (20 s each) at 1,000 mW/cm^2^. After cementation, all the samples were stored in distilled water for 24 h at 37 °C before the tensile bond strength tests were performed.

### Tensile bond strength test and fracture analysis

Tensile bond strength tests were performed for 13 specimens in each group using a load cell of 1 KN at a cross-head speed of 0.5 mm/min until failure using a Zwick/Roell Universal Testing Machine (Zwick/Roell, Ulm, Germany).

The TBS was expressed in MPa, and derived by dividing the force that was imposed (N) at the time of fracture by the bond area (mm^2^) (Tonial et al., 2010). After debonding, the dentin and ceramic surfaces were examined under an optical microscope at 20X to analyze the failure types. The failure types were classified as follows: adhesive (failure at the resin/dentine or resin/ceramic interface), cohesive (failure in the dentin or ceramic, or within the luting cement itself), or mixed (Rodrigues et al., 2015).

### Scanning Electron Microscopy (SEM) preparations and observations

For examination under SEM (Tescan VEGA3 LMU, Brno, CZ), the bonded teeth (*n* = 3) were cut mesiodistally by a low-speed diamond saw (IsoMet, Buehler, Lake Bluff, IL) under copious water. Then the dentin sides of all the samples were etched with H3PO4 for 5 s and washed with distilled water for 30 s. After that, the samples were immersed in sodium hypochlorite 2.5% for 3 min, then washed with distilled water for 30 s. The samples were then dehydrated in a graded series of ethanol solutions (Kharouf et al., 2020). All the specimens were analyzed using the SEM at an electron-accelerating voltage of 5 kV to assess the dentin/cement interface.

SEM observations for the dentin-resin interfaces were performed at different magnifications (800x, 3000x, 6000x). However, only one magnification (6000x) is demonstrated in the results of this study.

### Statistical analysis

Descriptive statistical methods were used for the statistical analysis, especially sample mean, standard deviation, coefficient of variation, and median. The normality of the data samples was tested using the Shapiro–Wilk test and homoscedasticity was tested using the Bartlett test. The Shapiro–Wilk test detected that the data of some groups were not normally distributed. Therefore, the Kruskal–Wallis test followed by a pairwise comparison using the Wilcoxon rank sum test were used to test the influence of dentin conditionings and different types of cements on bond strength and, in addition, to compare the median values among the groups (a = .05). The statistical analysis of the data was performed in R 3.6.2 (Vienna, Austria).

## Results

The tensile bond strength results among all the groups are summarized in Table 2. The highest mean value was observed for NX3. The bond strength obtained for MAX after the pre-treatment with PAA was the lowest mean value, and showed statistical differences from all the other groups (*P* < 0.05).

**Table 2:** Mean, Median TBS with (SD) for each luting agent after different dentin surface treatments. Upper letters indicate significant differences between the different groups (*p*  <  0.05).

Group	N	Mean (MPa)		Median(MPa)
NX3	13	9.66 ±4.53^a,b^		10.88
RLX-no	13	5.40 ±2.83^a,c^		5.62
RLX-PAA	13	5.73 ±3.01^e^		6.47
RLX-HPO	13	7.87 ±4.48^f^		7.20
MAX-no	13	5.36 ±3.25^f^		6.28
MAX-PAA	13	1.94 ±1.35^b−g^		1.98
MAX-HPO	13	7.58 ±4.81^g^		8.45

No statistical difference was found when MAX-no and RLX-no were compared.

The mean value of NX3 (the control group) was higher than that of RLX-no (*P* = 0.0488).

No statistical differences were found between RLX-HPO, MAX-HPO, and NX3 (*P* = 1.000). The dentin pre-treatment using phosphoric acid ameliorates the TBS of the RLX to be compared to NX3.

**Figure 1:**
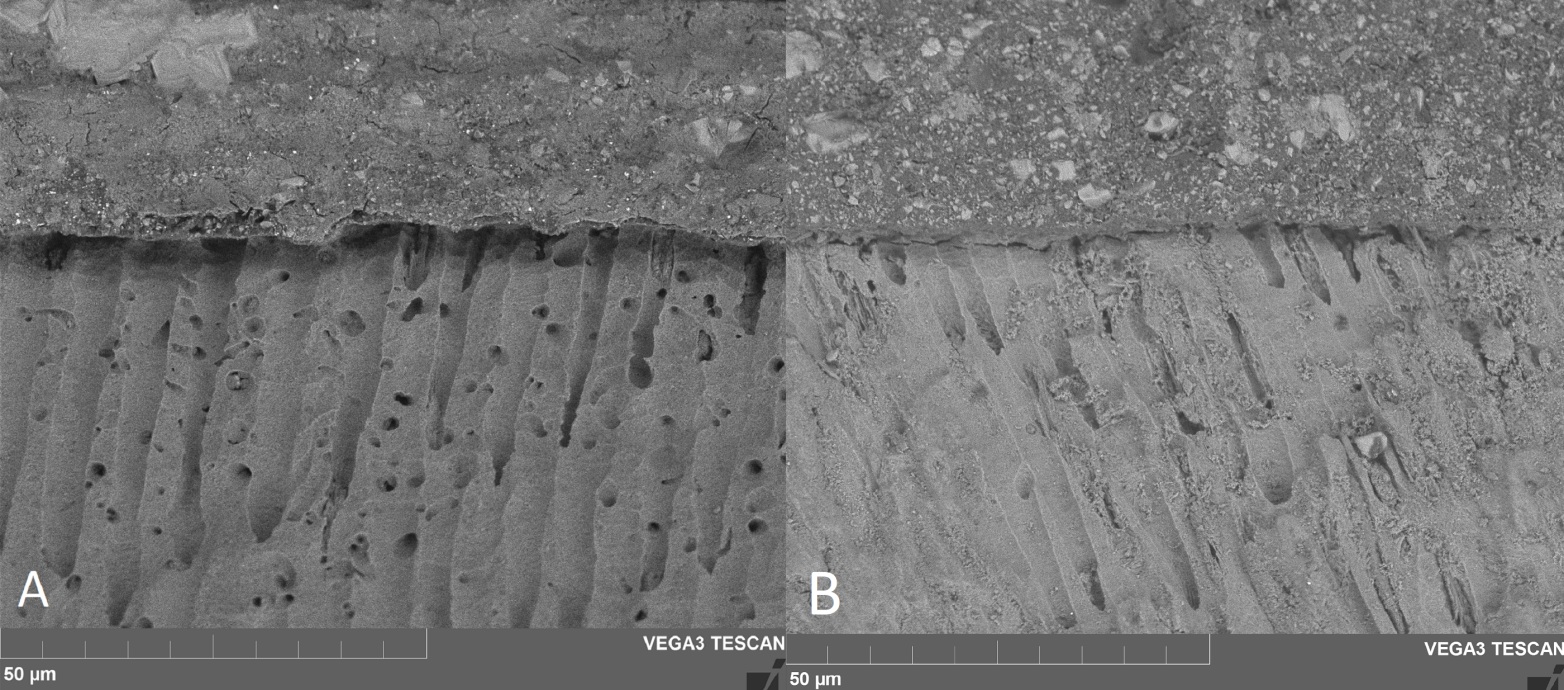
SEM observation (x6000 magnification). (A) Maxcem no-treatment, (B) Relyx no-treatment. SEM observations demonstrate no resin infiltration into dentin tubules when both self-adhesive cements were applied following manufacturer’s instructions.

**Figure 2:**
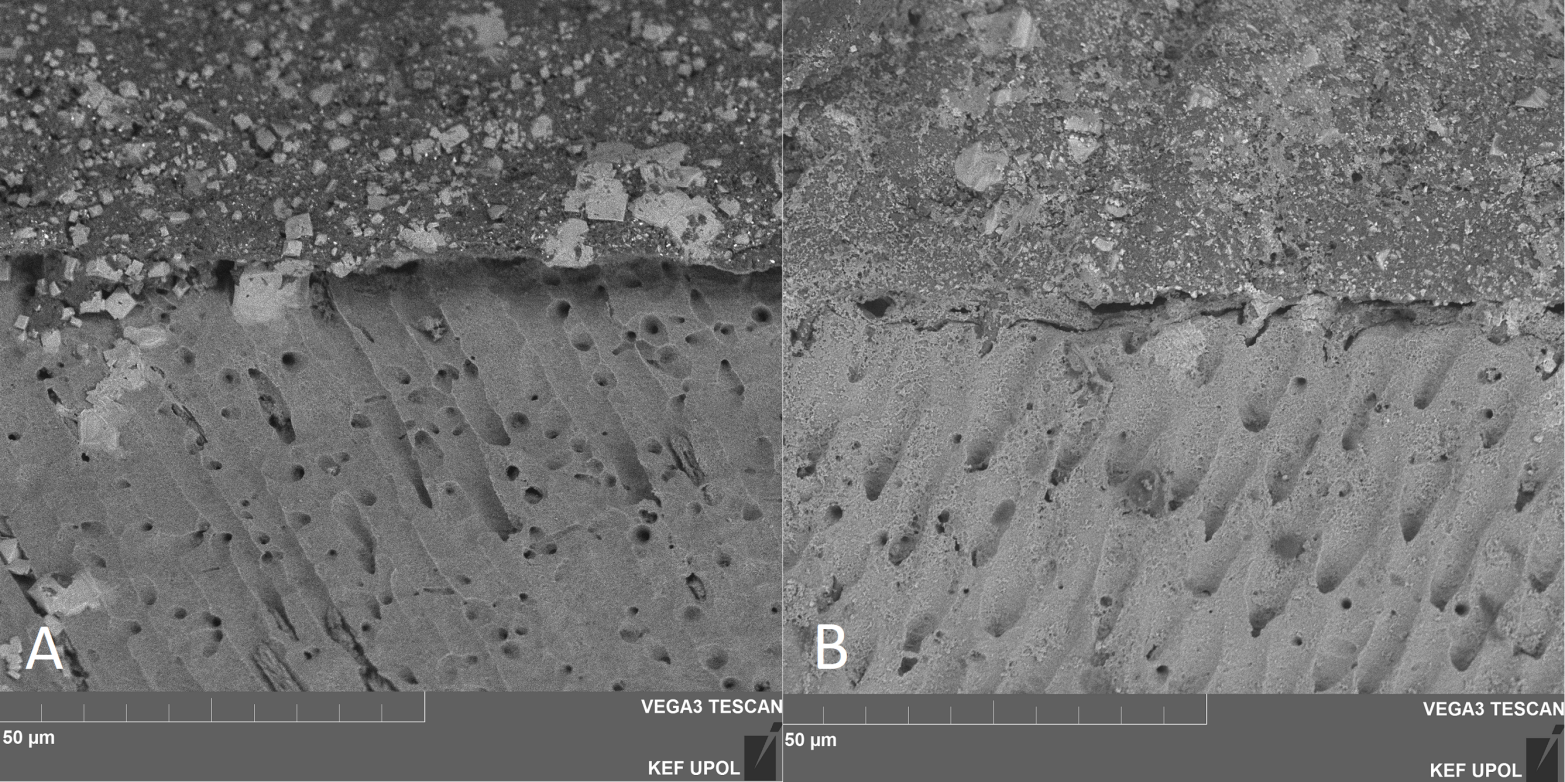
SEM observation (x6000 magnification). (A) MAX-PAA, (B) RLX-PAA. MAX-PAA demonstrated no-infiltration into dentinal tubules (A), whilst RLX-PAA presented a few short resin tag formation (B).

**Figure 3:**
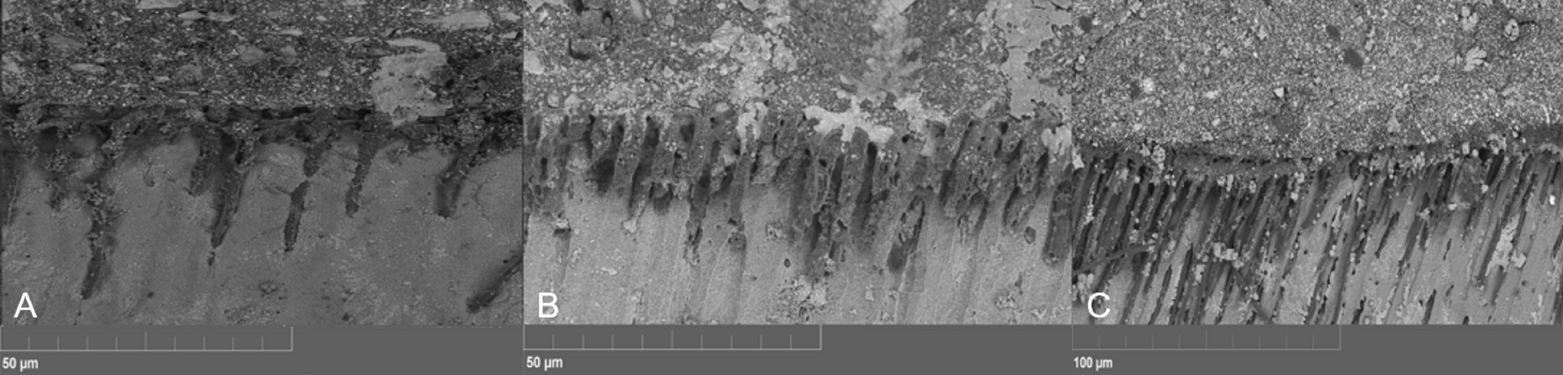
SEM observation (x6000 magnification). (A) Maxcem H3PO4, (B) Relyx H3PO4, (C) NX3. H3PO4-conditioned dentin interfaces showed well defined resin infiltration into dentin’s tubules and visible resin tags for all tested cements.

Concerning SEM observations, the cement/dentin interface for each pre-treatment with the cements that were used is illustrated in Figs. 1, 2 and 3. Only the etch-and-rinse groups (NX3, MAX-HPO, RLX-HPO) demonstrated the infiltration of resin tags into the dentinal tubules. In contrast, the groups with untreated dentin and/or with PAA pre-treatment showed no resin infiltration, except a few shorter tags with RLX-PAA.

Concerning the type of failure that was obtained after the TBS test, there were no cohesive or mixed failures among the no-pre-treatment groups. An adhesive failure was also dominant with the MAX-PAA group. Mixed and adhesive failures were observed in the MAX-HPO, RLX-HPO, RLX-PAA, and NX3 groups. The percentages of failure types are described in Table 3.

## Discussion

The existence of the smear layer has been recognized as the weak link in the bonding of selfadhesive cements to dentin (Santos et al., 2011). The smear layer contains dentinbuffering components that may participate in the neutralization effect during the setting of the selfadhesive resin cements (Stona et al., 2013). Clinically, the modification or removal of the smear layer is therefore necessary to form a hybrid layer to ensure a potent bond between the resin and dentin (Shinoda et al., 2011).

**Table 3:** Percentage of the failure mode: A- adhesive, M- mixed, C- cohesive.

	Relyx U200	Maxcem Elite Chroma	Nexus NX3 dual-cure
No pre-treatment	100% A	100% A	53.84% A, 46.15% M
H3PO4	69.24% A, 30.76% M	61.53% A, 38.46%M	
PAA	84.61% A, 15.38% M	100% A	

In the present study, when Maxcem was applied according to the manufacturers instructions (no pre-treatment), its bond strength was comparable to that of the EAR system (NX3) (*P* = 0.0778), while RLX had a lower statistical value (P = 0.0488). The self-adhesive resin cements, in general, have a limited ability to demineralize the hard dental tissues (Moda et al., 2018). However, the different chemical compositions of the self-adhesive cements could influence their mechanical properties and bonding performances (Hattar et al., 2015). This might explain the differences in behavior between the no-treatment groups (RLX and MAX) when compared to EAR. Similarly, Monticelli et al. (2008) showed that RLX had lower bond strength to dentin than EAR systems.

The application of polyacrylic acid as a pre-treatment with RLX did not significantly affect the bond strength values when compared to RLX-no. These results contradict the findings of Pavan et al. (2010), who verified a notable enhancement in the bond strength of this selfadhesive cement when 25% polyacrylic acid pre-treatment was performed for 10 s.

On the other hand, MAX with PAA pre-treatment had the lowest bond strength value when compared to the other groups. The present results of MAX-PAA are in accordance with the results obtained in the study of Mazzitelli et al. (2010). The authors found that the bond strength of a 2-hydroxyethyl methacrylate (HEMA)-based cement, such as Maxcem, which was used in our study, decreased significantly after dentin pre-treatment with PAA. Additionally, after the application of 25% polyacrylic acid (pH 1.53), the smear layer was partially removed, but all the tubules in the dentin stayed unplugged (Ayad, 2001). This type of demineralization could damage the interaction between the resin and the collagen in dentin (Mushashe et al., 2016). It is also suggested that monomers such as UDMA in Maxcem have hydrophobicity and thus might infiltrate less into dentin, even though UDMA has low molecular weight (Van Landuyt et al., 2007), which contributes to the lower viscosity of the cement. The differences in behavior between RLX and MAX after the application of PAA might also be because of the different chemical compositions (Hattar et al., 2015).

According to the dentin pre-treatment with H3PO4, the bond strength values of both selfadhesive cements (RLX-HPO and MAX-HPO) showed no differences from the EAR group. while there was a statistical difference between RLX-no and EAR groups. The enhancement of the bond strength with RLX after the etching of the dentin with H3PO4 may be explained by the fact that self-adhesive luting agents need an ionizing medium for the chemical reaction to get started, and after the tubules were opened by H3PO4, the hydration state of the dentin increased and optimized the acid/base reaction (Mushashe et al., 2016). In contrast, De Munck et al. (2004) showed that the use of H3PO4 as a pretreatment could reduce the bond strength values. They reported that the high viscous RLX was unable to reach the thick collagen mesh exposed by acid etching.

According to Maxcem, it has in its composition HEMA and GDM, which have one of the highest hydrophilicities among dental resins (Sokolowski et al., 2018). After the etching with H3PO4, the tubules in the dentin have been unplugged, thus such monomers might have the potential to infiltrate into the dentin tubules. The null hypothesis can be partially rejected, since the application of PAA reduced the bond strength with Maxcem, and the application of H3PO4 improved the bond strength with Relyx.

The findings of SEM observations on the surface of untreated dentin show no notable demineralization or real hybridization when compared to the groups treated with H3PO4.

The existence of resin tags in the dentinal tubules after the use of H3PO4 means that the tubule orifices were evident enough for resin to penetrate into the tubule and to hybridize with the nearby collagen fibrils, allowing better sealing (Sofan et al., 2017). Similar results were found in the study of Pisani-Proença et al. (2011). They reported that H3PO4 opened the dentinal tubules as a result of the removal of the smear layer, favoring the infiltration of the functional monomers within the self-adhesive cements into the dentin samples, consequently form a hybrid layer in addition to the resin tags.

In the present study, PAA pre-treatment was not able to open the tubules, and thus, no resin tags were formed except a few shorter tags with RLX-PAA. This study did not discuss the effect of the thickness of the cement layer or the tag length on the bond strength to dentin. However, Kharouf et al. (2021) demonstrated in their study that the thickness and the tag depth have no impact on the bond strength of a dental adhesive to dentin.

Concerning the type of failure that was obtained after the TBS test, with the no-treatment groups and MAX-PAA, there were no cohesive or mixed failures, whilst an adhesive failure was dominant. Mixed and adhesive failures were observed in the MAX-HPO, RLX-HPO, RLXPAA, and NX3 groups. The presence of mixed failures obtained for RLX-HPO and MAX-HPO is associated with the formation of resin tags, suggesting a greater interaction of the resin cement with dentin, which improves the micromechanical retention (Sofan et al., 2017).

The high variability of the values of bond strength reported in the published articles may reflect the lack of a standard testing protocol and the heterogeneity in tooth structure and composition. The relatively low TBS values obtained in this study might be due to the test type (macro-tensile bond strength) which was used (Van Meerbeek et al., 2010). he literature has not provided the minimum strength of the bond to the dental tissues that the luting agents must have in order to guarantee the longevity and success of the cemented restorations (Stona et al., 2013). Therefore, additional clinical investigations are needed before making any clinical recommendations.

One limitation of this study was that the tensile bond strength test was performed after 24 h of storage in water without aging. Therefore, further analysis should be performed in order to evaluate the impact of aging on the bond strength after different dentin pre- treatments. Another limitation of this in-vitro study is that usually, when the bond strength test is employed, the bonding performance is tested on a flat dentin surface, which does not mimic the oral environmental conditions.

## Conclusions

Within the limitations of this study, Maxcem Elite Chroma as a self-adhesive resin cement might be an effective alternative to conventional resin cement (EAR system). Etching the dentin with phosphoric acid does not have a negative effect on the bond strength of MAX to dentin. On the other hand, phosphoric acid improved the bond strength of Relyx U200 when compared to EAR cement. Polyacrylic acid did not affect the bond strength of Relyx U200 to dentin, whereas it significantly reduced the bond strength of Maxcem Elite. Therefore, we can also conclude that all self-adhesive cements cannot be classified as one homogeneous group.

## Supplemental Information

10.7717/peerj.11736/supp-1 Supplemental Information 1 The statistical analysis of the samples showing the methods which were used for the statistical analysis, especially sample mean, standard deviation, coefficient of variation, *p*-value, and median

10.7717/peerj.11736/supp-2 Supplemental Information 2 The values of tensile bond strength for each sample used in this study

## Additional Information and Declarations

### Data Availability

The following information was supplied regarding data availability: The raw measurements are available in the Supplemental Files.
